# The Evolution of the Druggist

**Published:** 1904-09

**Authors:** 


### TEXAS MEDICAL JOURNAL.

AUSTIN, TEXAS.

A MONTHLY JOURNAL OF MEDICINE AND SURGERY.

EDITED AND PUBLISHED BY
F. E. DANIEL, M. D.
Mrs. F. E. DANIEL, Managing Editor.

Published Monthly at Austin, Texas. Subscription price $1.00 a year in advance.
Eastern Representative: John Guy Monihan, St. Paul Building, 220 Broadway New York City.
Official organ of the West Texas Medical Association, the Houston District Medical Association, the Austin District Medical Society, the Brazos Valley Medical Association, the Galveston County Medical Society, and several others.

### THE EVOLUTION OF THE DRUGGIST.

### The Manufacturing Pharmacist the Doctor's Ally and Benefactor.

**Brief for Defendant.**

Sixty or seventy years ago, when there was usually one doctor in a village or community, and when medical colleges were few, far between and difficult of access—(those were "stage days)—a "doctor" began his studies under the old doctor who was on deck in that particular balliwick. He was "apprenticed" to the doctor, and chief of his duties was to grind up the crude drugs of the day, and to make powders, "boluses," tinctures, and syrups, etc., from them in a primitive fashion, an old iron mortar and pestle, and a dinner-plate for a pill tile and a few bottles and jars being his equipment. The doctor's *materia medica* consisted of gum opium, assafetida, "Peruvian bark" (cinchona), rhubarb, calomel and julap, salts and senna, ipecacuanha (it was then called), squills, blue mass, colocynth, castor oil, turpentine, tartar emetic, etc., and such indigenous medicinal plants as he and his cub boy could find in the gardens and woods, such as sage, balm, catnip, chamomile, skull-cap, willow bark, blackberry root, snake root, wild cherry bark, etc. The doctor dispensed his own medicine from saddle-bags. The boy was taught all the old doctor knew, in the course of some years, mostly empirical knowledge. He was taught that syrup or "vinegar" of squills, and of wild cherry bark were "good for" lung troubles, peruvian bark (before the days, even, of quinine) was "good for" chills; and in the course of time he was licensed by his preceptor to practice medicine. In time he, too, had a cub apprentice, and in time handed down to him the accumulated lore of "our noble profession." In larger towns and cities, and after awhile in villages, drug shops were opened, and the "apothecary," as he was called, did, on a little larger scale, what the doctor's cub had done; and in addition to selling patent medicines (as he does today, that being the bulk of his stock) and toilet articles, and soda water, as a side line he sold drugs and medicines and filled prescriptions. It became a fad to write a prescription, and it holds today, rediculous as it is sometimes made, by bad, nay, undecipherable writing or ignorance of the orthography of the ingredients. This was filled, if it could be made out, sometimes by a sleepy clerk, and now (sometimes) by a licensed pharmacist. A pill mass, say, as big as a pencil, and say, four inches long, was rolled out on a pill tile marked off in one-quarter inches, and the mass was divided by a questionable spatula into more or less equal parts, which were then rolled, one at a time, between finger and thumb, not always sterilized or even clean. Each pill may or may not contain the prescribed dose, say, of strychnine, for instance. In making up mixtures (for which an exorbitant price was and is now charged), if he can read the scrawl of the prescriber, and if he does not substitute "something just as good" as many, very many, do today, the patient may or may not get what the doctor intended him to get, and no one is ever the wiser. In Austin, to my knowledge, the aged mother of a leading physician took, by mistake, a teaspoonful of alleged tincture of aconite root, and it didn't "phaze" her.

This sort of thing is out of date. The dispensing druggist belongs to the last century, and should be relegated to the attic of antiquity, along with the spinning wheel and the hand-loom, the tallow candle and the coal oil lamp (the "light of other days"); the individual shoemaker and the small tailor—(the "Sninps" of the "Taming of the Shrew")—for pharmacy has kept pace with other industrial arts that supply the thousands of human needs, and not only are the staple drugs and chemicals and the officinal extracts and tinctures of the U. S. P. turned out by the ton, but mixtures of such ingredients and in such proportions as thousands of physicians have found efficacious, are made by machinery of precision into pills, granule, tablets, and mixtures, and furnished in any quantity and of definite (tested) strength and purity. The legitimate and honorable wholesale manufacturers of what are called proprietary medicines, also put up by the thousands, prescriptions which have really become staple, and the formula is printed on each package. The doctor has ready to his hand any combination he wants to use, in neat, condensed, and attractive form and of reliable quality and strength, and if he were wise he would oftener carry them and dispense them, *dispensing with* the dispensing druggist, too often a substitutor. He is a fossil of a past age. One reason, and a good one, why homœpaths are so popular, is, that when a patient dies or recovers, there is no big drug bill to pay, and two-thirds of the nauseous stuff left on hand. The drug bill is often larger than the doctor's, and when paid, sometimes, as has happened to yours truly, there is not money enough left in the family to pay the doctor. I say, the dispensing or prescription druggist is an anachronism; he is out of date. He stands in relation to the wholesale manufacturer of pharmacals as does the tallow candle or kerosene lamp to the splendid electric light; the stage coach to the lightning express.

And yet, there are those, amongst them the editor of the *Journal of the American Medical Association,* who decry the "commercialism of medicine," and condemn physicians for using proprietary medicines, "ready-made prescriptions," and ridicule them for "letting the pharmacist think for them." It is pointed out that 84 per cent of American physicians now prescribe proprietary medicines, and the lament is long and loud at the "encroachment'! I say it is right, sensible, proper, and legitimate, in reasonable bounds. Why laboriously write out, say, for instance, "℞. Chloral Hydrate, Gr. 60; Brom. Potas, Gr. 60; Ext. Can. Ind: ext. Hyoscy. *aa.,* Gr. 1; Aqua pura, ℥i. M. Sig.: Teaspoonful *pro re nata,*" and send it to a substitutor, lose two hours in getting it, at a cost to the patient of about three times its value, run the risk of substitution, or of getting too much or too little of the extract in a dose, when you could leave with the patient an ounce of Bromidia, and know that it is c. p. and reliable? I would not be thought partial to any one of the many reliable and elegant preparations, but I just take this well-known and popular article to illustrate my meaning.

The legitimate and honest wholesale manufacturers of those drugs and medicines most used, and which are as staple as cotton, and of the combinations of such drugs, in such proportions as experience has shown to be effective, are put upon the defensive, and I am here to defend them as the doctor's ally and benefactor. All honor to the intelligent, progressive, honorable, and honest men who have

"commercialized" and given us pure chloroform, ether, cocaine, morphine, the antitoxine serums, the hypnotics, antiseptics, hypodermic tablets, pure vaccine, grip tablets, laxative pills, reconstructives, all in elegant form and reliable, with the formula on every package! The doctor is not "letting the pharmacist think for him." That is the veriest rot. The pharmacist manufactures what the doctor needs in his business, and the intelligent doctor accepts the products of these great laboratories and uses them, just as sensible men now buy clothing, shoes, shirts, and other necessities, without having a measure taken and each garment made to order; just as he uses the electric light instead of the coal oil lamp. These preparations are labor-savers, time-savers (no waiting to wake the clerk at night and have a prescription filled); they are safer and more reliable, cleaner, and less expensive to the patient. It is up-to-date. All honor to the enterprising men who have made this possible! Millions of money are invested in plants that turn out proprietary medicines, many of which are now as staple as quinine, and big salaries are paid bright men to engage in the assaying, analyzing, and compounding of the drugs. It will not be long before, not 84 per cent, but 100 per cent, of the intelligent physicians will use such preparations—"ready-made prescriptions," as are of known merit, and they will carry them, at least to a great extent. They will cut loose from the risky prescription-filler,—he is a back number.

Draw, of course, a sharp line between the recognized and meritorious pharmacal preparations and proprietary articles, and the numerous "zones," "ines," and "ols," many of which are imitations of safe and reliable, popular and established articles having similar terminations. Select with discrimination. The reliable ones are known to progressive physicians who keep abreast of the progress of medicine and read the leading medical journals, for they are advertised to the profession only; they are his wares. And, of course, in this connection are not to be thought of, the villainous patent (secret) medicines (often fancy drinks of vile whisky) that every newspaper will tell you will cure anything on earth except death, and will stagger a mild case of that, and if you don't believe it, they cite you to the pictures which adorn the front page, of senators and generals galore, who testify to the wonderful virtues of these fakes and frauds. I have often wondered if many big men (and women) whose pictures adorn the papers are not paid for their testimonials. —for they are not all fools.

